# What Proportion Counts? Disaggregating Access to Safely Managed Sanitation in an Emerging Town in Tanzania

**DOI:** 10.3390/ijerph16183328

**Published:** 2019-09-10

**Authors:** Hans C. Komakech, Francis Moyo, Oscar Veses Roda, Revocatus L. Machunda, Kyla M. Smith, Om P. Gautam, Sandy Cairncross

**Affiliations:** 1 The Nelson Mandela African Institution of Science and Technology, Arusha 4222, Tanzania; 2 School of Civil Engineering, University of Leeds, Leeds LS2 9JT, UK; 3 WaterAid, London SE11 5JD, UK; 4 London School of Hygiene and Tropical Medicine, London WC1E 7HT, UK

**Keywords:** small towns, mapping, urban sanitation, access, SDG, Tanzania

## Abstract

Sustainable Development Goal (SDG) 6.2 sets an ambitious target of leaving no-one without adequate and equitable sanitation by 2030. The key concern is the lack of local human and financial capital to fund the collection of reliable information to monitor progress towards the goal. As a result, national and local records may be telling a different story of the proportion of safely managed sanitation that counts towards achieving the SDG. This paper unveils such inconsistency in sanitation data generated by urban authorities and proposes a simple approach for collecting reliable and verifiable information on access to safely managed sanitation. The paper is based on a study conducted in Babati Town Council in Tanzania. Using a smartphone-based survey tool, city health officers were trained to map 17,383 housing units in the town. A housing unit may comprise of two or more households. The findings show that 5% practice open defecation, while 82% of the housing units have some form of sanitation. Despite the extensive coverage, only 31% of the fecal sludge generated is safely contained, while 64% is not. This study demonstrates the possibility of using simple survey tools to collect reliable data for monitoring progress towards safely managed sanitation in the towns of global South.

## 1. Introduction

The United Nation’s Sustainable Development Goal (SDG) 6.2 designates 2030 as the “finish line” for low-income countries to “achieve access to adequate and equitable sanitation and hygiene for all” [1]. It also aims to end open defecation and pays special attention to the needs of women and girls including people in vulnerable situations [2]. Since 2015, the race to meet this goal has seen an increase in governments’ eagerness to gather sanitation information to inform national policies and interventions [3]. Some experts, however, see the SDGs to be an overly ambitious target for many African countries. The critics point at the lack of human and financial capital to fund sanitation investments and limited state capacity to collect reliable information required to measure success and monitor progress as the main impediments for African countries to achieve Goal 6.2 [2].

In Tanzania, for example, most towns do not have reliable baseline data on access to sanitation facilities and their sustained use. The information that is available is fragmented and cannot easily be verified. Therefore, attempts to achieve universal access to adequate and equitable safely managed sanitation by 2030 might be derailed by a lack of reliable data needed to organize and design targeted interventions. Often the data being gathered by local authorities are presented only to show overall success but not where bottlenecks exist. This is mainly due to techniques of data collection and the local officials’ vested interest of only showing improvement in overall sanitation coverage. The local authorities focus on collecting information about absence or presence of a toilet (user interface i.e., the superstructure slab and pan), or the visible aspect, with no detailed information on the type of containment (storage) or what happens downstream in the sanitation service chain. As a result, there is a dearth of well-disaggregated sanitation data that can be used to inform the design of targeted interventions needed to make progress towards achieving the SDG target of universal access to safely managed sanitation across the country.

This study was designed to provide evidence-based findings that will facilitate the planning and selection of viable intervention options for improved management of the entire sanitation service chain in a small town of Tanzania. The study is based on the mapping of sanitation facilities in eight wards of the Babati Town Council, Tanzania. A simple mobile phone survey tool was developed and used to collect data on access to sanitation services in the town. To track the country progress towards the SDG sanitation goal, the Ministry of Health is implementing the National Sanitation Campaign (NSC). The ministry has developed a registry system that is being used by local government health officers to collect sanitation data in the area of jurisdictions. By comparing the two methodologies of sanitation data collection and mapping, we aim to identify the disparities in data reliability and validity, and to unveil what really counts towards achieving SDGs relating to sanitation

The challenges in data collection together with the differences in classification of sanitation facilities means that reports of success or failure cannot be compared across nations, and sometimes across towns. The key question remains therefore, what proportion of safely managed sanitation counts? Our attempt to find an answer to this question prompted us to develop a survey tool for use in the freely available tool and software, Open Data Kit (ODK), installed on an android smartphone. We engaged local government health and executive officers to carry out the data collection exercises in their areas of jurisdiction within the Babati Town Council. The ODK software allows for the storage of big data sets that can be easily aggregated/consolidated and retrieved for analysis, and easily accessible for independent verification. By collecting information throughout the sanitation service chain, the survey offers an opportunity for disaggregation of access to sanitation and for setting a realistic and verifiable baseline information. Although our efforts in one town may not be conducive to generalization, the development of a smartphone-based tool to generate easily verifiable data is a major contribution of this of this study. We show how a simple mapping tool (using open source software and low-cost smartphones) and engaging town council staff to collect households sanitation data (household and housing unit used interchangeably in this paper) can lead to the collection of reliable sanitation data and contribute to tracking progress towards the SDG targets. Integrating this tool into national campaigns, such as the National Sanitation Campaign (NSC) of Tanzania, can help in tracking progress towards the SDG targets. This technique coupled with growing mobile network coverage and lower costs of smartphones and internet connection provides opportunity for governments to collect and aggregate reliable and verifiable sanitation data at a relatively low cost. 

## 2. Review of Key Concepts and Definitions on Safely Managed Sanitation

During the Millennium Development Goals (MDGs) era (1990–2015), progress towards meeting the sanitation target was initially monitored using the binary category of either improved or unimproved facilities [4]. According to the MDG definition, an improved sanitation facility separates human excreta from human contact. The unimproved, which includes shared sanitation facilities on the other hand, comprises of facilities that were considered to put users at risk of being in contact with human excreta [4]. The binary approach was later modified to a service ladder comprising three rungs: unimproved, shared, and improved sanitation. Yet in this new classification, the focus remained more strongly on technology types. Improved sanitation includes facilities that were connected to sewer, septic tank systems, pour-flush latrines, ventilated improved pit, and simple pit latrines. Public sanitation facilities were categorized as shared and unimproved, and included open pit latrines and bucket latrines.

**Figure 1:**
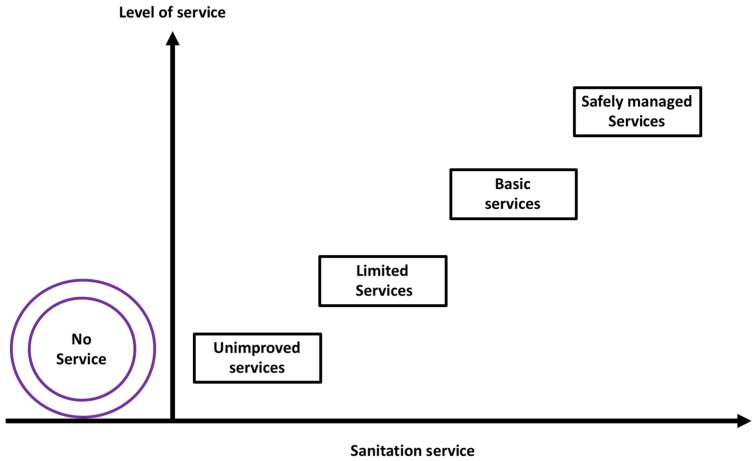
Joint Monitoring Program sanitation service ladder.

However, the technology-based categorization of sanitation services has been critiqued as being biased towards some of the technologies. For instance, promoters of composting and urine-diverting toilets which were not in the list felt excluded [5]. The Joint Monitoring Program (JMP) have since refined the sanitation classification and adopted a modified version of the sanitation ladder to monitor and report progress towards the SDG 6.2 [4]. Presently, SDG 6.2 uses normative definitions of sanitation targets and indicators, putting an emphasis on the proportion of the population using a safely managed sanitation service. Improved sanitation facilities include flush/pour-flush to piped sewer, septic tank, or pit latrine; composting toilet or pit latrine with slab. Safely managed sanitation is defined as “the use of improved sanitation facilities which are not shared with other households, where excreta are safely disposed in situ or temporarily stored then emptied, transported, and treated off-site or transported through sewers to a wastewater treatment facility”. Safely managed sanitation is a new addition, at the top level of the JMP sanitation service ladder (see the representation in Figure 1). As one moves from left to right on the sanitation axis (x-axis), the costs, and level of service also increases for households.

In addition, for monitoring progress towards the SDG, the JMP sanitation ladder has been modified to include: no service (open defecation), unimproved service, limited service, basic service, and safely managed services (Figure 1). No service or open defecation includes disposal of human excreta on fields, forest, bushes, open bodies of water, beaches, or other open spaces or with solid waste. Unimproved refers to the use of pit latrines without a slab or platform, hanging latrines, and buckets. Limited is when improved sanitation facility is shared by two or more households. Basic service on other hand is the use of improved facilities that are not shared by other households. Safely managed which sits at the top of the ladder indicates the use of improved facilities that are not shared with other households and where excreta are safely disposed in situ or transported and treated off-site (see SDG 6.2).

Based on the above definitions of different service levels under SDG 6.2, it is important to focus the debate on what type of sanitation technologies are included in the different service levels. Some scholars argue that monitoring types of technologies defined as improved is an imprecise proxy for the quality of the services [2]. According to Kvarnström et al. [5] and Mara [6], a function-based sanitation ladder is a more appropriate way of measuring and monitoring success. The classification of shared sanitation used by more than one household as limited has also sparked a lot of debate. The main JMP argument for excluding shared sanitation facilities in the improved category is that it increases the risk of adverse public health outcomes. Arguably, households relying on shared sanitation are more prone to acute diarrhea, helminths, etc. [7]. However, other scholars argue that the evidence on health challenges associated with shared sanitation is weak due to many reasons: the diverse typologies of shared sanitation facilities; uncertain methodologies often used for measuring health risks; and lack of evidence regarding actual latrine use, distance, waiting time, and cost. Major differences in many study designs also limit comparability between cases [7].

According to Evans et al. [8] and Mara [6], the classification of shared sanitation as limited is also a disincentive for public investment in unplanned areas or slum sanitation. Feasible sanitation investment in such areas is likely to be related to improving or building new shared facilities which will not be counted as progress towards the SDG safely managed sanitation target [8]. As a result, more public attention is now geared towards fecal sludge management (FSM) and sewer networks that only benefit planned areas and more affluent urban communities. Focusing on technologies appropriate only in planned and more affluent areas risks creating or reproducing inequalities in sanitation service provision, which is contrary to the SDG human right principle of leaving no-one behind. Therefore, we argue that safely managed sanitation should only serve as an ideal standard that every country or town should aspire to, but should not side-track policy makers from the provision of sanitation services that allow households to put their feet on the first rung of the sanitation ladder to reduce access inequality [8].

In addition to the types of sanitation facilities, the SDG sanitation service ladder requires a shift in the way progress was being monitored and reported by the JMP. The new service ladder allows for a disaggregated analysis of the sanitation services being provided. Although the use of representative samples to measure access has been questioned, long term monitoring data collected for the MDG through the national census, national demographic health surveys (DHS), and United Nation Children’s Fund (UNICEF) multiple indicator cluster surveys (MICS) studies are available to track the first three rungs of the service ladder (unimproved, limited, basic). Yet, at a country level, the classification of sanitation services is sometimes quite different from those used by the JMP, which complicates the calculation of the global statistics. For instance, in Tanzania, sanitation facilities are classified as unimproved, improved, basic, or safely managed. Where improved sanitation facilities include any non-shared toilet of the following types: flush/pour-flush toilets to piped sewer systems, septic tanks, and pit latrines; ventilated improved pit (VIP) latrines; pit latrines with slabs; and composting toilets [9].

## 3. Materials and Methods 

### 3.1. Study Area

**Figure 2:**
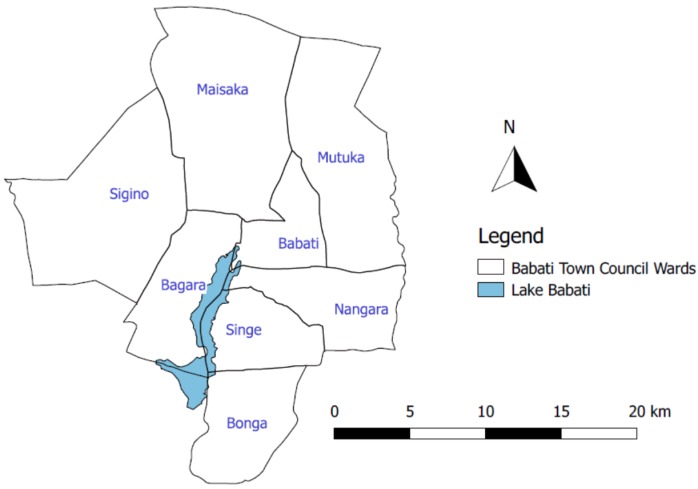
Babati Town Council administrative boundary.

The study was conducted in Babati Town, located in Manyara region, Northern Tanzania. The town is situated at about 168 kilometers south of Arusha city and 700 kilometers from Dar es Salaam. Babati Town is located at the northern end of Lake Babati catchment area, a tourist hotspot. Babati Town covers an approximate area of 460.86 km^2^ (Figure 2). The town was upgraded and accredited with town council status in 2014 following the division of Arusha region into the two regions of Manyara and Arusha. The secession of Manyara from Arusha region compelled the central government to upgrade at least one area in the newly established Manyara region to township status to become a regional headquarter. Administratively, Babati Town has eight wards comprising of 36 streets (urban area) and 13 villages (peri-urban area), with a total population of approximately 93,108 residents (NBS, 2012).

Babati Town’s population growth is estimated at 3.2% per year (above the national average), which currently means the town population could have reached above 108,000 individuals. The decision of the Tanzanian Government in 2015 to move government offices and ministries from Dar es Salaam to Dodoma, positioned Babati Town as a central place for people travelling to the capital from Tanga, Kilimanjaro, and Arusha regions, which are in the northern part of the country. This stimulates growth of business such as hotels, lodges and street vendors (locally referred to as machinga). The population growth and business development, however, also come with increased production of fecal and solid wastes in the town. 

### 3.2. Data Collection Method

This study used a survey methodology with a research design aimed at obtaining an overall picture of the sanitation situation in the small town. It employed both quantitative and qualitative methods to collect sanitation information along the service chain. The study target aimed to reach every housing unit in Babati Town (total enumeration) and managed to collect information from 17,383 out of the estimated 20,000 housing units (approx. 87% of the official records of the town dwellings). Ethical approval was obtained for this research from the National Institute for Medical Research (NIMR) in Tanzania. The term housing unit is used here to signify that not all visited homes were for a single domestic dwelling but also that one sanitation facility may be used by two or more households (e.g., housing complexes developed for renting). The discrepancy between housing units visited for data collection and estimated number of households in Babati may also be due to the fact that i) there is no updated list/number of dwellings in Babati; ii) some dwellings were not occupied at the time of this research; and iii) security restrictions exist for some housing for police and prison staff quarters. A structured questionnaire was developed in XLSForm format and then converted to Open Data Kit (ODK) XForm for use on android-based smartphones and tablets. The ODK software and step-by-step guide on how to develop the tool is available for free online [10]. The questionnaire tool used in this study can be provided to anyone needing it upon request to the lead author. The survey collected data included GIS location, ward, street, gender of owner, education of owner, user interface, containment, year of construction, number of users, sanitation outlet, emptying mechanism, and open defecation, etc. Local government officials working at the ward and street/village level, whose job responsibilities also include collection of sanitation data, were trained on how to use the data collection tools. This was in the form of a two-day training conducted to agree on common terminologies used to identify different components of the sanitation services chain and to reduce errors. A practical field survey was also conducted to test the functioning of the survey tool outside the study area.

In total, 56 local government officials were involved in the data collection, visiting about 20 to 100 dwellings per day depending on the terrain and distance between housing units. In each housing unit, the sanitation facility (user interface and visible parts of the containment) was georeferenced and photographed using the Geographical Positioning System (GPS) and camera embedded on the smartphones or tablets. The tools were programmed to automatically save the GPS readings when the accuracy is within zero to four meters. The survey questionnaire was also programmed in such a way that the enumerators could move to the next question only when the current active cell was filled with valid information. Respondents were residents of the dwellings who were above 18 years of age, knowledgeable with the dwelling sanitation design, construction, use, and management. In the case where a respondent was not certain of some of their responses, phone calls were made to other residents of the dwelling for clarification.

The use of local officials who have the responsibility to collect sanitation data and have legal access and power to inspect dwellings in the areas of their jurisdiction increased the study potential to reach almost all the dwellings in the town. Since the study was action research, involving the local authorities was a critical component as well as strengthening their capacity in generating high quality data needed for making decisions on sanitation services. Our aim was to strengthen existing personnel and systems that will remain in place for the long term, both to ensure sustainability and to increase data reliability and quality. In terms of training officials on how to appropriately engage with households, we note that it is important to work with the local leaders (street chairmen and ten house cell leaders) to build trust of the community in the process. The GPS records and photos reduced the chances of those whose sanitation facilities or practices were not legal to withhold information. It also closed loopholes for enumerators (who are supposed to have sanitation data in their offices) to duplicate shelved information or fill the questionnaire from their offices. In-depth interviews and stakeholders’ meetings were conducted to validate data from the sanitation mapping. Respondents for the in-depth interviews included Babati Town Council (BTC) and Babati Water and Sanitation Authority (BAWASA) staff, selected residents, and all enumerators involved in the data collection. The interview was used to validate household survey data especially on issues such as open defecation, lack of toilets and “vomiting of toilets”. Vomiting of toilets is a local term used to indicate the practice of digging a hole next to a full pit latrine and diverting the sludge to this hole. Respondents were from individuals with a wide knowledge of the town, sanitation service providers, or regulators. This helped the study to have a complete and accurate picture of the types of toilets existing across Babati Town.

### 3.3. Data Management and Analysis

In total 17,383 housing units were surveyed and mapped. Data collected was imported into Microsoft Excel and cleaned to generate sanitation maps and descriptive statistics. Excel pivot tables were used to group and compare the data on various types of sanitation interfaces, containment, outlet, emptying, transport, and treatment. The QGIS 3.8.1 “Zanzibar” was used to visualize and analyze the spatial configuration of the sanitation facilities in Babati Town. Qualitative information from in-depth interviews were grouped into themes following their similarities or differences to support and qualify quantitative information.

## 4. Results

### 4.1. Settlement Descriptive Statistics

Out of the 17,383 housing units surveyed, 56 were offices, hotels, churches, and mosques among others (Table 1). Based on the survey, a total 109,397 people were reported as accessing sanitation from the mapped 17,383 housing units. The majority (71%) of housing units were owned or under the care of individuals with primary level education (81% male and 19% female). Overall, 82% of the housing units had some form of sanitation facility.

### 4.2. Distribution of Sanitation Technology Types

In this section, the sanitation data presented are from the National Sanitation Campaign (NSC) and this study’s sanitation mapping exercise. The two data sets were collected by the same local government officials but using different tools. Based on the 2017 Babati Town NSC data, only 0.3% of the dwellings did not have sanitation facilities, 7.9% had traditional latrines, and most of the households were reported to have improved latrines (Table 2). Traditional latrines are categorized as unimproved because they are almost all dilapidated, normally built of a few wooden poles, grass, cloth, or plastic materials. The pits are less than four meters deep; the floors are not well covered and fecal matter can easily be seen. The idea that most households are using improved sanitation of some kind had the town authority start planning for a town sewer network and treatment lagoons. When Babati Town NSC data are converted to the categories of the JMP sanitation ladder, it shows that 49.6% of the dwellings are using pit latrines (improved and unimproved) and 30.0% have VIP latrines. Also, only 20.1% of the sanitation facilities in Babati Town can be classified as flush latrines of all types. Since no information is collected on the containment, emptying, and treatment the NSC data cannot be used to compute the proportion of the population accessing safely managed sanitation in the town. However, the user data from this study indicates that about 35.4% of the houses have traditional latrines, 15.7% uses improved pit latrines, 28.2% uses flush latrines of all types, 2.4% have VIP latrines and 4.5% practice open defecation (Table 2).

There is a great difference in the VIP data (30% in the NSC and 2.4% from the survey). This is likely because of the difficulties of identifying ventilated improved latrines faced by the health officers and data collectors for the NSC registry. Before training, we noted that all ward health officers were unable to correctly identify the different sanitation user interfaces. For instance, one health officer defined traditional sanitation as “choo cha muda” meaning short-term-use latrine. It is, therefore, possible that after proper training coupled with practical field visits, the local authorities engaged were more likely to correctly distinguish VIP latrines from the other types of facilities. 

**Figure 3:**
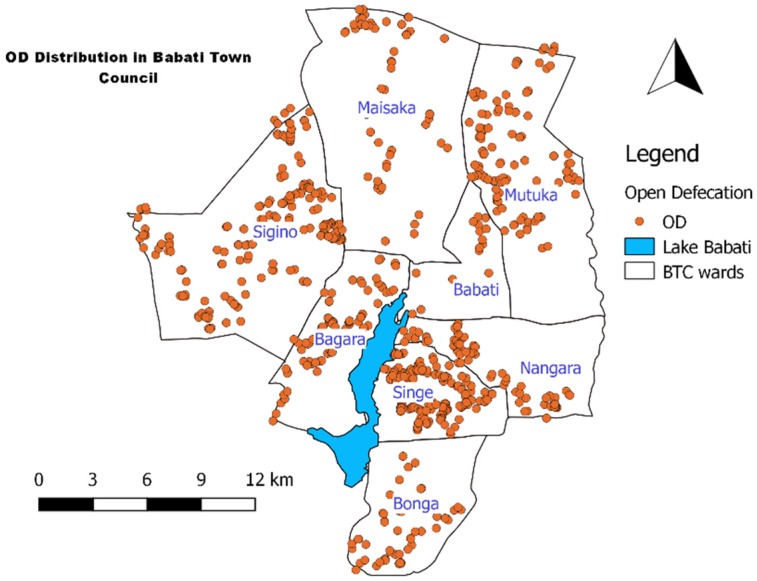
Distribution of households practicing open defecation in Babati Town.

Further analysis of the 14,199 housing units with some form of sanitation revealed that 10% of the containment are septic tanks, and 7% sealed tanks, while 20% were properly covered and then abandoned when full (Table 3). However, only 1% of sanitation facilities are reported to be emptied when full, and the emptied sludge is either disposed of onsite or transported to open land dedicated for fecal sludge discharge by the town authority. The site is close to cultivated food crops. About 4.5% of the housing units surveyed practice open defecation (calculated based on the average number of users per housing unit size of about 7.7 this is roughly 6000 people). Open defecation was not reported in the NSC data but from this study, it is practiced in all 8 wards of the town (Figure 3), hence posing health risks to the whole Babati Town population. 

From the study, 80% of the facilities were reported as not yet full or as not being full since construction, about 18% are not emptied, only 1% of the facilities are emptied and another 1% not known.

**Figure 4:**
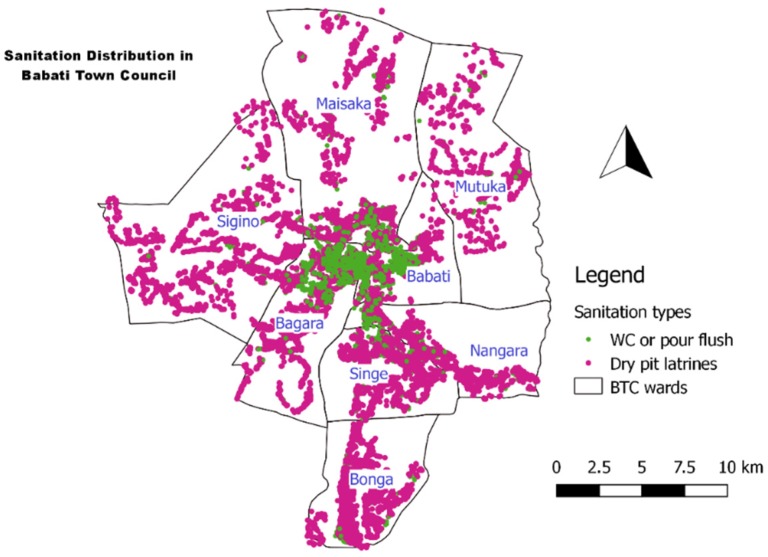
Distribution of dry and wet sanitation in Babati Town Council.

From the data collected during this study it is possible to prepare maps of distribution of types of sanitation user interfaces and containment. To show that Babati is not ready for a central sewer system, we classified the sanitation facilities into dry and wet sanitation (Figure 4) and used it to inform the town sanitation planning process which was being carried out at the time of the research. The classified map was also used during a sanitation scenario planning exercise also conducted as part of a wider piece of research. As a result, the local authority has selected to implement fecal sludge management in the town, and a consultant will be hired to develop the business plan.

### 4.3. Access to Safely Managed Sanitation in Babati Town

To estimate the proportion of safely managed sanitation in Babati Town, we used the fecal waste flow diagram methodology. The fecal waste flow diagram, popularly known as the Shit Flow Diagram methodology (SFD), is an approach that graphically visualizes the efficiency of fecal sludge management of an area [11]. It is a useful approach for tracing the flow path of human excreta along the sanitation service chain: containment, emptying, transport, treatment, and final disposal or reuse.

From the survey data, the proportion of households with access to different sanitation containment is summarized in the SFD matrix (Table 4). The proportion for each type was derived by counting the containment in each category. As shown in Table 4 there are also septic tanks or pour-flush connected to soak pits or pit latrines with high risk of groundwater contamination. The following assumptions were made to develop the SFD for the town:

a) Assumed that 50% of pits/tanks are in areas with high risk of groundwater contamination.
b) Assumed 10% of sealed tanks, pits, septic tanks are being emptied.
c) Visual inspection of locations of each sanitation categories on the groundwater contour maps, location of 435 deep and shallow wells constructed by the households [12].
d) Assumed that open defecation derived from housing units is the same when converted to proportion of the town population practicing open defecation.

The assumptions were validated through field visits to public and private toilets, interviewing households with shallow wells, and review of findings from a groundwater contamination study carried out in the town [12]. There is no central sewerage network or central treatment plant in BTC but only a dedicated place where fecal sludge is discharged by vacuum truck. In terms of open defecation, we rounded up to 5% of the population that still practice open defecation.

The risk of groundwater pollution can be estimated from data on drinking water from groundwater sources, hydrogeology and the distance between groundwater sources and sanitation facilities (as indicated in assumption “c” above). The risk assessment tool from the SFD Graphic Generator guides the user to select the appropriate sanitation option in the selection grid (either located in low or high-risk areas of groundwater pollution). Both assumption “a” (50% of pits/tanks are in areas with high risk of groundwater contamination) and the visual assessment of wells from the study carried out in the town on groundwater contamination [12] were used in developing the SFD graphic to show the risk of groundwater pollution. 

**Figure 5:**
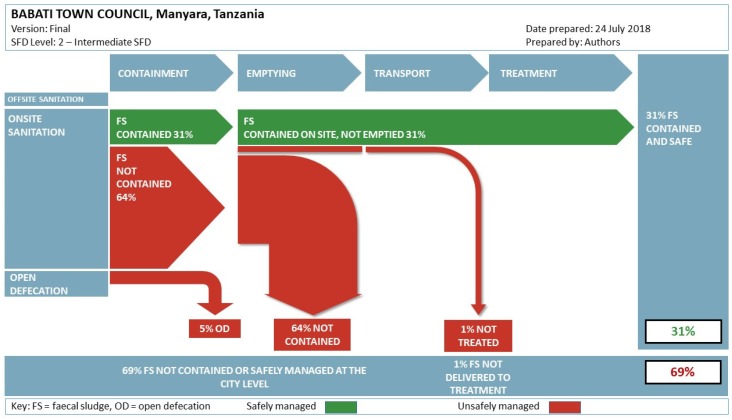
Babati Town fecal sludge flow diagram.

Using the matrix in Table 4, we developed the Babati Town Shit Flow Diagram (SFD). It shows that although sanitation coverage in Babati Town is high (e.g., about 82% of the housing units had some form of sanitation); only 31% of the fecal sludge currently produced is safely contained on site, not emptied (i.e., safely managed), while about 69% is not contained (Figure 5). Out of the 69%, about 1% of the fecal sludge is emptied but it is discharged untreated, while 64% is not contained on site and 5% is open defecation (note that SFD tool currently round up decimal places leading slightly higher total). The NSC classification only focuses on the user interface where investment is done by dwelling owners and, therefore, the information cannot be used directly to develop the fecal sludge flow diagram. Although the concerted efforts being made through the Tanzania National Sanitation Campaign are allowing households to put their feet on the first rung of the sanitation ladder, which reduces access inequality [13], it is not possible to determine the proportion of safely managed sanitation in the town. The fecal waste flow diagram for Babati was used to change the mindset of the local authority and consider selecting fecal sludge management as the best option for the town in the short to medium term.

### 4.4. Disparities between Reported NSC Data and Sanitation Mapping Exercise

The rapid “urbanization” of Babati Town, makes it an interesting and peculiar case for understanding and disaggregating access to safely managed sanitation in emerging towns in the countries of the global South such as Tanzania.

This town-wide sanitation mapping study reveals large disparities between the data collected in this study compared with the sanitation data reported by local authorities. It was revealed that the methodology we have adopted in this study produces more information than the routinely used NSC registry-based methodology. The officers who were involved during the data collection are responsible for enforcing environmental regulations and encouraging residents to adopt improved sanitation facilities. The town-wide sanitation mapping exercise has uncovered that the local authority’s methods, tools/technology, type of data collectors, and categories used to define and collect sanitation data are not robust and have some limitations. The current NSC sanitation reports are based on data collected through paper-based surveys with no clear methods for data verification. In 2017, the Babati Town NSC data show that 50.4% of households have improved sanitation facilities, about 9% of the households have hand-washing facilities, and five streets/villages have full sanitation coverage. However, through this study, where all data points were georeferenced and photographed, the number of households with improved sanitation facilities is less than half of what is in the Babati Town’s NSC database, for instance, open defecation is practiced in all wards.

### 4.5. The Potential for Replication of This Study Methods

In this study, we surveyed the entire town, something which is not possible to accomplish in large cities. To promote the comprehensive survey of the sanitation service chain, it is important to consider what type of sampling could be feasible and applicable for large urban towns. We tested the potential of systematic random sampling of housing units in a town for sanitation mapping. The sampling strategy we used to get the number of housing units for further analysis was calculated based on the following formula [14]:

(1) $$\mathrm{Sample}\;\mathrm{size},\;n=\frac{N}{1+N{\left(e\right)}^{2}}$$

Whereby “e” is the level of precision (%), “N” is the total number of housing units, and “n” is the sample size for survey. A precision level of 5% was selected in order to get optimal sample size (recommended “e” is between 5% and 10%) [15]. The sample size for a town with 17,383 housing units is then 391 units. To get the housing list, a unique code was assigned to the full list of housing units (17,383) in Excel. Then in an empty column the formula "=rand ()" was used to generate a random number for each data point. The data table was then sorted in ascending order on basis of the random numbers. The randomized order was used to select the first 391 housing units for analysis. Table 5 and Table 6 shows that it is enough to use a representative sample to estimate sanitation user interface coverage, the percentage for the different categories are nearly the same. Critical to the use of representative sample is of course the local capacity to generate an accurate list of housing units within an area. Once the survey tool is designed to capture information on user interface, containment, emptying, treatment, and final disposal or reuse and budget is allocated, it is possible to realistically estimate the proportion of access to safe sanitation that counts.

## 5. Discussion

In Babati, the consensus had been that sanitation coverage was over 90%, meaning that the authorities are only dealing with the “last mile”, basically that eliminating open defecation in the town can now be achieved. So, the local authorities had the believe that there was no open defecation in Babati town before the survey was started. From our total sanitation mapping in Babati Town, most user interfaces are connected to containment systems that are rarely, if ever, emptied. Most users reported that they will simply construct a new pit once the old one is full and that open defecation is practiced by a small proportion of the population throughout the town. Building a new pit when one is full is of course only a short-term solution, it is not a sustainable at the city level where, eventually, there will be no longer be space available to build new pits in the future. In Babati Town, as in other towns and cities in Tanzania, limited data are being generated on the entire sanitation service chain. We can state that the current focus on user interface may be leading local authorities and the government into counting and using incomplete data on safely managed sanitation service provision.

The observed large proportion of user interface and containment types on the lower rungs of the JMP sanitation ladder could also be attributed to the priorities of the National Sanitation Campaign (NSC) that is being carried out in Babati Town Council. The NSC registry captures five types of user interfaces; traditional pit latrine, improved traditional pit latrine, VIP, pour-flush, WC flush, and no sanitation or whether feces are visible around the household surroundings. The focus, therefore, is only on ensuring that people have some form of sanitation that can be measured and without considering the entire service chain. By focusing only on the user interface, the authority may be over counting the number of people with access to safely managed sanitation services in the town. This is because any form of pour-flush sanitation facility is automatically considered an improved sanitation facility and yet feces may be discharged to rivers or open channels. 

The other challenge for small towns is when a town’s status is upgraded, it also impacts the way sanitation is seen by the new town authorities. In the case of Babati, the process did not follow steps stipulated in the local government act/regulation where a rural area shall first be upgraded to a trading center, a small-town authority, and thereafter become a town or city. The bureaucratic growth process to some extent provides space for development of infrastructure needed to cope with the socio-economic services needs of a town setting, and for people to change their mindset through social learning and invest in safe sanitation. As a result, Babati Town has grown to have diverse sanitation types, with a large proportion of dwellings with no access to safely managed sanitation. Taking into consideration the fact that the SDGs pledge to “leave no-one behind”, and specifically in goals 6.1 and 6.2 on universal access to sanitation; it becomes apparent that Babati Town’s attempts at achieving these goals will need extra effort. This is a challenge because moving up the ladder from the lowest possible sanitation type is a slow process [16]. In addition, about 14% of households in Babati Town are dependent on their neighbor’s sanitation facilities. However, this type of access to sanitation can be categorized as neighbor-shared access and improved [16]. Further scrutiny of access to shared facilities between neighbors at late night hours, when owners are not present at the dwelling or when considering issues of cleanness and proper use [13], reveals how complex the situation is and open defecation might become inevitable.

The disparities between sanitation data reported by local authorities and those revealed by this town-wide sanitation mapping study, demonstrate a major challenge for poor countries to achieve the SDG target on sanitation. The town-wide mapping exercise has uncovered that local authority’s methods, tools/technology, type of data collected, or categories used to define and collect sanitation data have limitations. Babati Town’s NSC report, for example, indicates that five streets/village have full sanitation coverage, implying zero open defecation in some streets/villages. However, this is contrary to the data collected using a digitized method, where GPS points and photos were recorded. Both sets of data were collected by the local authority’s officers including the wards/streets executives, health officers, and community development officers. Despite the same local officers getting involved in the two different exercises, the data generated are different. The differences come from the method of data collection used such as manual filling of forms in the NSC study versus the use of a mobile phone-based survey tool. The NSC data are collected by enumerators under the supervision of village or ward health officials who have limited budget allocated to facilitate their work. In some cases, this lack of budget may lead to lower motivation levels to collect the data. In other cases, the officers may also have vested interest to report improvement. The problem is compounded by the fact that there are no clear methods used by the authorities for data verification. One critical challenge for NSC data validation is lack of money. It costs about USD 500 per year to print NSC registry books for small towns such as Babati (personal communication). The annual budget designated for sanitation mapping per town varies from USD 6000 to USD 15,000 out of which only about USD 2000–5000 may be allocated for the NSC survey. About USD 13,000 is required to collect data from 17,383 housing units, this is roughly paying USD 8–10 per day to each enumerator. However, the local authority usually pays USD 4 per day to their enumerators, which is comparatively low. It is still possible, however, to use the same NSC resources to conduct comprehensive sanitation surveys. The methodology used in this study can be made at a reasonably low cost and is easily scalable to other cities with different sanitation options especially if a careful random selection at street level is done. 

Moreover, apart from issues of tools and human biases, categories used to define sanitation facilities are important. The Shit Flow Diagram (SFD) generated using the town-wide sanitation mapping exercise, reveals that currently the local authorities are not considering the proportion of safely managed sanitation. The main questions any sanitation intervention must strive to answer, therefore, are: what is the basis for lumping certain types of sanitation facilities into a certain sanitation category? Does the category clearly reflect the full sanitation service chain? The NSC classification, for example, groups sanitation facilities into five categories, namely unimproved traditional toilets, improved traditional toilets, VIP latrines, toilets that use water and ecological toilets. These categories do not portray any information about the containment type, and do not disaggregate access through the sanitation service chain. The NSC emphasis is on the “political face” of sanitation service, the user interface, where users can easily associate the health risks to the direct human contact with excreta. It is important, therefore, to focus on understanding what proportion of safely managed sanitation counts, or, simply, what benefits, success, and/or failure is defined or embroiled in the sanitation categories. Nevertheless, it is not only the proportion of safely managed excreta that is important, but it is also important to assess if the sanitation service delivery in town is sustainable in the medium to long term. Building a new pit when the existing pit is full may not be a sustainable solution for households even if the fecal sludge is safely contained on site.

Similarly, the SDGs and the shift towards considering the full sanitation chain are still quite recent and governments have yet to catch up. This necessitates the need for sanitation interventions such as the NSC to break away from old thinking and approaches that employed politically motivated sanitation categories, where governments focused on implementing policies or projects to fit their political agenda and claim political credits. The NSC categories do not consider the potential for groundwater contamination [12,17] and include interventions that have very limited or no budgetary pressure on the government. The focus on the user interface where investments are largely the responsibility of a dwelling’s owner, allows local authorities to excuse themselves from their key role as the providers for public services. In addition, without aggregating the data throughout the sanitation service chain it will be difficult for the local authority to measure real progress or for residents to hold them accountable. Clear descriptions of sanitation categories are an important entry point for planning interventions to reduce or eliminate sanitation related challenges such as fecal contamination of underground water used by poorer urban households and Water, Sanitation and Hygiene (WASH) associated diseases. This, together with increased capacity for data collection, consolidation, analysis, and interpretation will facilitate governments to track inequalities in safely managed sanitation. Additional work is required to understand the relationship between inequalities in different elements of safely managed services, so that these can be more systematically monitored in future reports for growing small towns such as Babati.

For the sake of discussion, there is room for cost saving (money and time) when a clear sampling strategy is applied using an accurate list of housing units within an area. For the National Sanitation Campaign, the local authority currently collects data on a quarterly basis with the objective of reaching every household at the end of the year. As stated above, selecting a good sample size can produce the same result and track progress of a community as a whole. However, it may not be seen by the local authority as an appropriate method for monitoring the progress of each household along the sanitation ladder. These would be important discussions to hold with the government authorities if they were to consider scaling up the use of this data collection technique.

### Limitations and Implications

The main limitation for this study was the tension of BTC being a project partner and at the same time a regulator of the sanitation sector in the town. The latter resulted in a small number of people who did not have toilets to disappear during the visit or quickly build new toilets after getting information about our survey from friends or relatives. Data on the numbers of respondents who did not agree to answer the survey or that built new toilets are not reported in this paper. This challenge was minimized by training enumerators (the local government officers) not to punish people during the mapping exercise. During the research, an effort was made to provide information about the survey and help household members to feel comfortable when approached by enumerators. Also, a small number of toilets were not observable as they were located inside bedrooms. Finally, mapping all of the housing units in a town requires time and financial resources for trainings, testing the survey tool and analysis of data for informed decision making. In Babati, 56 officials were trained and were able to visit 20 to 100 dwellings per day. This study design is feasible (in terms of time and budget) for small towns such as Babati, but it is unlikely to be feasible for large cities, for instance a city of 4 million people such as Dar es Salaam. However, the local authorities can still easily integrate the research methodology and the tool used in their data collection programs. In Tanzania, NSC data are being collected on a quarterly basis by enumerators at the street level and it is, therefore, possible for this tool to be integrated in their routine. At a national level, the approach provides an opportunity to engage in policy discussions regarding monitoring and planning for citywide sanitation services.

## 6. Conclusions

The commitment at the core of the SDGs to “leave no-one behind” is the most ambitious commitment governments have made on access to sanitation to date. A key question going forward is to understand what proportion of safely managed sanitation counts towards the SDG for sanitation in a given country. As it stands now, the indicators and data used by JMP are based on national and local records and databases, which are not consistent and are difficult to verify. Leaving no-one behind requires access to credible data and information. Lack of such valuable information on sanitation leads to poor planning and prioritization of investment by local authorities. A small town in Tanzania, just like many other small towns in the countries of the global South, often dreams of a network sewer with advanced wastewater treatment. Sewer systems are often not the right investment choice for small towns in low-income countries in the short to medium term (10–20 years planning period), specifically given the low number of sanitation facilities that could connect to a sewer system. In the short to medium term, small towns, such as Babati, should prioritize harmonization of sanitation designs, supervision of construction, providing training to artisans, enforcement of sanitation bylaws, and demarcation of clear areas for future construction of sanitation infrastructure. The town’s authorities can also invest in a small number of decentralized wastewater treatment systems. Adopting a phased approach towards city-wide sanitation services is the best option for small towns such as Babati.

Our Babati study is likely one of the first comprehensive sanitation mapping carried out in Tanzania. The data collected serves as a baseline and can be used to develop a sustainable database for sanitation improvement and contribute to appropriate urban planning for sanitation services. We have shown how a simple mapping tool (using open source software and cheap smartphones) and engaging town council staff to collect data can lead to the collection of reliable sanitation data. Integrating this tool into national campaigns, such as the National Sanitation Campaign (NSC) of Tanzania, can help in tracking progress towards the SDG targets. Moreover, the growing mobile network coverage, and lower costs of smartphones and internet connection means that it is possible for governments to collect and aggregate sanitation at a relatively low cost. However, we must note that the politics of data and knowledge will always be an issue in reporting progress towards the SDGs. The proportion of what is considered safe sanitation is likely to remain a subject of debate in countries where these phone-based mapping results may contradict existing records. For the local authorities, whatever happens beyond the user interface is like the adage “out of sight, out of mind”. Yet, sanitation is a public good with the health benefits to households are gained only when everyone has access [18,19].

## Figures and Tables

**Table 1:** Statistics of housing units surveyed in Babati Town Council.

**Housing Unit Characteristics**	(*n* = 17,383)	%
Dwellings	17,327	99.7
School	6	
Church	22	
Mosque	15	
Office	3	
Market	1	
Hotel	4	
Absent (no-one was around)	5	
**Gender of Owner/Head of dwelling**	(*n* = 17,327)	%
Female	3257	19
Male	14070	81
**Education Owner/Head of dwelling/responsible**	(*n* = 17,383)	%
Don’t know	388	2
No formal education	1534	9
Primary education	12,319	71
Secondary education	2299	15
Tertiary education	843	5
**Age of Owner/Head of dwelling/responsible**	(*n* = 17,383)	%
Unknown	244	1
18–21	231	1
21–30	1927	11
31–40	4532	26
41–50	4485	26
51–60	3064	18
Above 60	2900	17
**Housing unit with sanitation**	(*n* = 17,383)	%
Yes	14,199	82
No	3184	18

**Table 2:** Distribution of sanitation facilities as per the JMP sanitation service ladder (source: NSC database and this study).

Babati Town NSC Data			Sanitation Mapping		
Type of Sanitation Facility	Number of Households	Percent (%)	Number of Households	Percent (%)	Remarks
WC/pour-flush toilets	3956	20.1	4900	28.2	WC/pour-flush
VIP latrine	5901	30.0	425	2.4	VIP
Traditional pit latrines	1551	7.9	6152	35.4	Most houses had traditional pit latrine. These are still pit latrines
Improved traditional pit latrines	8210	41.7	2722	15.7	These are still pit latrines
Ecological sanitation					Not identified
Open defecation	No data	No data	786	4.5	Bushes, gardens, drains, etc.
Without Sanitation/share	65	0.3	2344	13.5	Households using neighbor’s sanitation
Not identified			54	0.3	Only access sanitation for 54 houses not identified
Total houses	19,683		17,383		

**Table 3:** Types of sanitation containment.

Containment Type	Number	Percent (%)
Septic tank	1451	10.2
Sealed tank	988	7.0
Lined pit but open bottom	483	3.4
Lined pit but semi-permeable walls and open bottom	2487	17.5
Unlined pit	5765	40.6
Pit, properly abandoned when full /properly abandoned	2991	21.1
Don’t know	34	0.2
Total	14,199	100.0

**Table 4:** Estimates of Sanitation Containment Matrix for fecal sludge flow diagram.

Containment Type	Estimated Proportion of Population Using This Type	Estimated Proportion of This Type That Is Emptied
Septic tank with soak pit	8	10
Sealed tank with soak pit	12	10
Lined pit, open walls, and bottom but no overflow	11	10
Unlined pits, no overflow	15	10
Open defecation	5	
Pit of all types, never emptied but abandoned and covered with soil no overflow	3	
Septic tank connected to soak pit but with high groundwater risk	7	10
Sealed tank connected to soak pit but with high groundwater risk	11	10
Lined pit, open walls, and bottom but with high groundwater risk	11	10
Unlined pits with high groundwater risk	14	10
Pit of all types, never emptied but with high groundwater risk	3	

**Table 5:** Comparison of sanitation user interface.

User Interface	Random Sample		Complete Mapping	
	Number of Households	Percent (%)	Number of Households	Percent (%)
WC/pour-flush toilets	109	27.9	4900	28.2
VIP latrine	8	2.0	425	2.4
Traditional pit latrines	134	34.3	6152	35.4
Improved traditional pit latrines	75	19.2	2722	15.7
Open defecation	12	3.1	786	4.5
Share	52	13.3	2344	13.5
Not identified	1	0.3	54	0.3
**Total (households)**	391	100	17,383	100

**Table 6:** Comparison of sanitation containment.

Containment Type	Random Sample	Percent (%)	No. Full Mapping	Percent (%)
Septic tank	29	8.9	1451	10.2
Sealed tank	36	11.0	988	7.0
Lined pit but open bottom	3	0.9	483	3.4
Lined pit but semi-permeable walls and open bottom	60	18.4	2487	17.5
Unlined pit	120	36.8	5765	40.6
Pit, properly abandoned when full /properly abandoned	78	23.9	2991	21.1
Don’t know	29	8.9	34	0.2
**Total**	391		14,199	
